# Supplementation With *Spirulina platensis* Improves Tracheal Reactivity in Wistar Rats by Modulating Inflammation and Oxidative Stress

**DOI:** 10.3389/fphar.2022.826649

**Published:** 2022-05-31

**Authors:** Aline de F. Brito, Alexandre S. Silva, Alesandra A. de Souza, Paula B. Ferreira, Iara L. L. de Souza, Layanne C. da C. Araujo, Bagnólia A. da Silva

**Affiliations:** ^1^ School of Physical Education, University of Pernambuco, Recife, Brazil; ^2^ Post-Graduation Program in Physical Education UPE/UFPB, Recife, Brazil; ^3^ Physical Education Department, Health Sciences Center, Federal University of Paraiba, João Pessoa, Brazil; ^4^ Federal University of Tocantins, Licentiate in Physical Education, Tocantinopolis, Brazil; ^5^ Postgraduate Program in Natural and Synthetic Products Bioactive, Health Sciences Center, Federal University of Paraiba, João Pessoa, Brazil; ^6^ Department of Biological Sciences and Health, Roraima State University, Boa Vista, Brazil; ^7^ Department of Biophysics and Physiology, Institute of Biomedical Sciences, University of São Paulo, São Paulo, Brazil; ^8^ Pharmaceutical Sciences Department, Health Sciences Center, Federal University of Paraiba, João Pessoa, Brazil

**Keywords:** respiratory mucosa, lipid peroxidation, inflammatory response, prostaglandins, epithelium

## Abstract

Spirulina platensis has shown effectiveness in the treatment of allergic rhinitis in rats, but its action in tracheal reactivity or on markers of relaxation and antioxidant profile has not yet been possible to determine. In this paper, the animals were divided into the groups healthy (SG) and supplemented with S. platensis at doses of 50 (SG50), 150 (SG150), and 500 mg/kg (SG500). We also evaluated nitrite levels, lipid peroxidation, and antioxidant activity through biochemical analysis. For contractile reactivity, only SG500 (pEC50 = 5.2 ± 0.06 showed reduction in carbachol contractile potency. Indomethacin caused a higher contractile response to carbachol in SG150 and SG500. For relaxation, curves for SG150 (pEC50 = 5.0 ± 0.05) and SG500 (pEC50 = 7:3 ± 0:02) were shifted to the left, more so in SG500. We observed an increase in nitrite in the trachea only with supplementation of 500 mg/kg (54.0 ± 8.0 µM), also when compared to SG50 (37.0 ± 10.0 µM) and SG150 (38.0 ± 7.0 µM). We observed a decrease in lipid peroxidation in the plasma and an increase in oxidation inhibition for the trachea and lung in SG150 and SG500, suggesting enhanced antioxidant activity. S. platensis (150/500 mg/kg) decreased the contractile response and increased relaxation by increasing antioxidant activity and nitrite levels and modulating the inflammatory response.

## Introduction

Allergic diseases of the respiratory tract such as asthma and rhinitis are an inflammatory state in which clinical symptoms such as shortness of breath, wheezing, and tightness in the chest result from either an increased limitation of expired airspace or bronchial hyperreactivity involvement (Fahy, 2015). In this inflammatory context, reactive species are produced, which can cause oxidative stress, resulting in physiological changes in the epithelium and the airway wall structure, in smooth muscle cells and the immune system, leading to the pulmonary limitations characteristic of these diseases (Lambrecht & Hammad, 2015).

The use of antioxidant products shows promise in the treatment and prevention of respiratory tract diseases. In this context, natural products of *Spirulina platensis*, filamentous cyanobacterium, have been used as a food supplement by humans for many years due to the high nutritional values in *Spirulina platensis*, such as high protein content, amino acids, minerals, and antioxidants. Besides, it has shown hypolipidemic, hypoglycemic, and antihypertensive properties and antioxidant and anti-inflammatory activities (Appel et al., 2018).

Spirulina has shown effectiveness in the treatment and prevention of allergic rhinitis in rats. This treatment acted by reducing inflammatory reactions and the mastocyte number in the nasal mucosa (Chen et al., 2005). As well as improved symptoms of rhinitis in humans (Nourollahian et al., 2020).

Tracheal reactivity has been an experimental model used for investigations that aim to analyze some aspects related to asthma or other allergic diseases of the respiratory tract that present contractile hyperresponsiveness of the tracheal rings (Montaño et al., 2018). The Food supplementation of *Spirulina platensis* proved itself effective in preventing hypercontractility in other smooth muscle models, uterus (Revuelta et al., 1997), aorta (Brito et al., 2018), corpus cavernosum (de Souza et al., 2018) and ileum (Araujo et al., 2020).

Despite the significant evidence on the importance of *S. platensis* in the inhibition of inflammatory markers, so far, this investigation first aimed to analyze the pattern of contraction and relaxation of the health trachea rings to the administration of different doses of S. platensis and to analyze the involvement of the prostanoids and nitric oxide metabolites and lipid peroxidation in health Wistar rats. We study has shown the influence of this cyanobacterium on the response of health trachea rings or relaxation markers and antioxidant profiles.

## Materials and Methods

We followed the methods of Brito et al. (2018; 2019; 2020).

### Substances

Glucose (C_6_H_12_O_6_), monobasic potassium phosphate (KH_2_PO_4_), hydrochloric acid (HCl), and magnesium sulfate heptahydrate (MgSO_4_.7H_2_O) were obtained from Nuclear (Brazil). Sodium chloride (NaCl) was purchased from Dinâmica (Brazil). Potassium chloride (KCl), calcium chloride dihydrate (CaCl_2_.2H_2_O), and sodium bicarbonate (NaHCO3) were acquired from Vetec (Brazil) and acetylcholine chloride (ACh) from Merck (Brazil). Phenylephrine (PHE) was obtained from Pfizer (United States) and aminophylline and indomethacin from Sigma Aldrich (Brazil). Ethylenediaminetetraacetic acid (EDTA) (1:250) was purchased from BioTécnica-Advanced Biotechnology (Brazil) and carbogen mixture (95% O_2_ and 5% CO_2_) from White Martins (Brazil). The weighing of the substances was done with a GEHAKA analytical balance model AG 200 (Brazil).

### Preparation and Supplementation With Spirulina platensis

*S. platensis* in lyophilized powder form was acquired from Bio-Engineering Dongtai Top Co., Ltd. (Nanjing, China) (Lot No. 20130320). the Pharma Nostra Quality Control Laboratory (Anapolis-GO, Brazil) (Lot No. 1308771A) analyzed a sample for certification that the extract was obtained from S. platensis. Then, Dilecta Manipulation Drugstore (João Pessoa-PB, Brazil) (Lot No. 20121025) prepared the used S. platensis. *S. platensis* was dissolved daily in saline in proportions of 0.005, 0.015 and 0.05 g/ml for the preparation of doses of 50, 150 and 500 mg/kg, respectively., which were administered to the animals at the end of the preparation. The preparation of each dose of S. platensis was performed daily on all supplementation days. Supplementations were performed for a period of 8 weeks for all doses (50, 150 and 500 mg/kg/day). The supplements were administered for 8 weeks (adapted from Juarez-Oropeza et al., 2009) with oral administration daily (between 12 and 2 p.m.), using stainless steel gavage needles (BD-12, Insight, Ribeirão Preto-SP, Brazil) and 5 ml syringes with 0.1 ml precision (BD, Higilab, João Pessoa- PB, Brazil).

### Animals and Experimental Protocol

For the experimental animal model, the subjects were male Wistar rats (Rattus norvegicus), weighing between 250 and 300 g, from the Prof. Thomas George Bioterium of the Research Institute for Drugs and Medicines from Federal University of Paraiba (UFPB). The animals were weighed on a GEHAKA semi-analytical balance (São Paulo-SP, Brazil). In order to guarantee the reliability during the measurement of the weight of the same, the scale had a cylindrical tube of appropriate size to place the animal, which was effective to contain its movement. All the animals were maintained on a nutritionally balanced feed (Labina^®^) with free access to water, with controlled temperature (21°C), and a 12 h light-dark cycle (lights on at 6 a.m.). All experiments were conducted between 8 a.m. and 8 p.m., according to the guidelines for the ethical use of animals (Sherwin et al., 2003). The experimental protocol was previously approved by Ethics Committee in Animal Use (CEUA/UFPB) with certificate number 0511/13.

The animals were divided into saline (0.9% NaCl, control) and supplemented with *S. platensis* (50, 150, and 500 mg/kg) groups. Thus, the study was composed of the following groups with 20 rats randomly divided into a saline group (SG, control) and groups supplemented with *S. platensis* at 50 (SG50), 150 (SG150), and 500 mg/kg (SG500). For the reactivity experiments, the sample of each result included five different experiments (n = 5) and for the biochemical experiments we used (n = 10). Each preparation was from different animals. The selected sample size was chosen to avoid using an excessive number of animals, and this number provides enough reliability for the results obtained.

### Concentration-Response Curves

#### Pharmacological Evaluation of Spirulina platensis on Tracheal Reactivity

The rats treated with saline or *S. platensis* at the end of the intervention protocols, the animals were anesthetized with ketamine 100 mg/kg (i.p.) and xylazine 10 mg/kg (i.p.), followed by a complementary method with were euthanized by cervical dislocation, followed by cervical vessel section. This method was adopted since the tissue to be evaluated in the experiments would be completely damaged if euthanasia were performed by guillotine, in addition to the injury that would be caused to the epithelium of the organ, mechanisms that were also evaluated in our study. The animals’ thorax was open and dissected. Their tracheas were carefully removed and cleaned to separate them from all connective and adipose tissue. Then, we separated tracheal segments containing three to four cartilage rings. In addition, lungs were removed for further dosing of oxidative stress. After the removal, the lungs were cleaned in a Krebs solution to remove blood residues, placed in Eppendorf, and stored in a -80°C freezer until analysis.

For isometric responses, the tracheal segments were suspended through stainless steel rods in organ baths (6 ml) containing Krebs solution under a tension of 1 g, maintained at a temperature of 37°C, and remained in rest for 60 min gassed with carbogen. The nutrient solution was renewed every 15 min to prevent interference of metabolites during all the experiments (Sherwin et al., 2003).

After a stabilization period, a tonic contraction was induced by 10^−6^ M Carbachol (CCh). The integrity of the tracheal epithelium was verified by the addition of arachidonic acid (AA) to the organ bath at the concentration of 10^−4^ M during the tonic phase of the first response induced by CCh. In this case, the rings that showed relaxation equal to or greater than 50% were considered with intact epithelium and relaxation equal to or less than 10% without epithelium [18]. The protocols used both the rings with and without the functional epithelium.

### Effect of Spirulina platensis on Cumulative Contractions Induced by Carbachol

The procedures quoted in the previous item were followed by the preparation and assembly of the rat tracheal rings. After examination of the epithelium, the preparations were washed every 15 min for 30 min. After 30 min, a CCh-induced cumulative curve (10^−9^–10^−3^ M) was performed in all groups for further comparison [18]. The contraction was expressed as the percentage of contraction induced by CCh. The negative logarithm of the drug concentration, which produced a half-maximal response (pEC50) value, was obtained from the concentration-response curves obtained on rings with and without functional epithelium of the saline solution or treated with *S. platensis* groups. The efficacy parameters were evaluated through maximum effect (Emax), also under the same conditions.

### Effect of Spirulina platensis on Relaxation Induced by Aminophylline

After a stabilization time, a tonic contraction was induced with 10–6 M CCh, and aminophylline (AMF) (10^−10^ × 10^−2^ M), a non-selective phosphodiesterase inhibitor [19], was cumulatively added to the organ bath in the groups treated with saline solution or *S. platensis* to induce the relaxation of the preparation.

Relaxation was expressed as the reverse percentage of the contraction induced by CCh. The potency parameter was evaluated by pEC50 values from the concentration-response curves obtained in rings with and without functional epithelium of the groups treated with saline solution or *S. platensis*. The efficacy parameters were evaluated through Emax, also under the same conditions.

Evaluation of the prostanoid signaling pathway in the relaxing action of Spirulina platensis in rat trachea: Effect of Spirulina platensis on CCh-induced tonic contractions in the absence and presence of indomethacin.

After the procedures previously cited but before obtaining the CCh induced contraction in trachea rings with and without functional epithelium, 10^−5^ M indomethacin, a non-selective inhibitor of the enzyme cyclooxygenase (COX), was added in independent preparations (Juárez-Oropeza et al., 2009). To ensure the non-interference of prostanoids resulting from COX action, after 30 min, a cumulative curve to CCh (10^−10^–10^−3^ M) was performed in the presence of indomethacin (INDO) in all groups for further comparison (Tschirhart et al., 1987).

The contraction values were expressed as the percentage of the contraction induced by CCh. The pEC50 values were compared in the absence and presence of indomethacin from the concentration-response curves obtained in the rings of the samples from groups treated with saline solution or *S. platensis*. The efficacy parameters were evaluated through Emax, also under the same conditions.

### Biochemical Measurements

After the animals were euthanized, the blood was collected by cardiac puncture (Okafor et al., 2011) and placed in test tubes containing anticoagulant (EDTA) to obtain plasma for quantification of nitrite, malondialdehyde (MDA), and antioxidant activity (Ohkawa et al., 1979; Brand-Williams et al., 1995; Juárez-Oropeza et al., 2009). The samples were centrifuged at 1207 g for 15 min using a Centribio 80-2B-15 ML centrifuge (Guarulhos-SP, Brazil). The plasma was transferred to Eppendorf tubes and stored at -20°C until analysis. The trachea fragments 8 mm long were quickly removed, cleaned with Krebs solution to remove residual blood, and stored inside Eppendorf tubes in a freezer at -80°C until the analysis of the MDA markers, antioxidant activity, and nitrite.

### Nitrite Assessment in Plasma and Trachea

Nitrite concentrations were determined by the Griess method as described by Green et al. (1982). Accordingly, the Griess reagent was prepared using equal parts of 5% phosphoric acid, 0.1% N-1-naphthyl ethylenediamine (NEED), 1% sulfanilamide in 5% phosphoric acid, and distilled water. Then, 500 μl volume of plasma or tissue macerate was added to 500 μl of Griess reagent, followed by absorbance reading at 532 nm after 10 min. The blank used was 100 μl of the reagent plus 100 μl of 10% potassium phosphate buffer. The sodium nitrite (NaNO_2_) standards were made by twofold serial dilutions to obtain: 100, 50, 25, 12.5, 6.25, 3.12, and 1.56 mM solutions. A Biospectro SP-220 spectrophotometer (Curitiba-PR, Brazil) was used for absorbance readings.

### Assessment of Lipid Peroxidation

To identify possible changes in the lipid matrix of cell membranes, which results from the indirect effect of the production of reactive oxygen species, we used the oxidant activity technique of measuring thiobarbituric acid reactive substances (TBARS) with lipid hydroperoxides (Okafor et al., 2011). For this, tissue samples (trachea and lung) were homogenized with 10% KCl in 1:1 proportion. Then, tissue homogenate and plasma samples (250 μl) were incubated in a water bath at 37°C for 60 min. After that, the samples were precipitated with 400 μl of 35% perchloric acid and centrifuged at 26,295 g for 10 min at 4°C. The supernatant was transferred to new Eppendorf tubes, and 400 μl of 0.6% thiobarbituric acid was added, followed by incubation at 95–100°C for 30 min. After cooling, the samples were read at 532 nm. MDA concentration in plasma and tissue samples was determined using an MDA standard curve constructed using a standard solution (1 μl of 1,1,3,3-tetramethoxypropane in 70 ml distilled water) diluted in a series of 250, 500, 750, 1000, 1250, 1500, 1750, 2000, 2,250, 2,500, 2,750 and 3,000 μl of distilled water. In the tissue, the absorbance values were normalized to the dry weight present in each sample volume.

### Evaluation of Antioxidant Activity

This procedure was based on the method described by Brand-Williams et al. (1995), where 1.25 mg DPPH (2,2-diphenyl-1-picrylhydrazyl) were dissolved in 100 ml of ethanol, kept under refrigeration, and protected from light (aluminum paper or amber glass). Then, a volume of 3.9 ml of DPPH solution and 100 μl of the tissue homogenate or plasma was added to appropriate centrifuge tubes. The tubes were vortexed and allowed to stand for 30 min. They were centrifuged at 13,416 g at 20°C for 15 min, and the absorbance of the supernatant was read at 515 nm. The results were expressed as the percentage of the inhibition of oxidation, where: antioxidant activity (AOA) = 100 - ((DPPH • R) T/(DPPH • R) B 100), where (DPPH • R) and (DPPH • R) B correspond to the concentration of DPPH • remaining after 30 min, measured in the sample (T) and blank (B) prepared with distilled water.

### Statistical Analysis

The functional results obtained were expressed as the mean and standard error of the mean (S.E.M.), while the biochemical results as mean and standard deviation (S.D.). These results were statistically analyzed using one-way analysis of variance (ANOVA) followed by Bonferroni’s post-test, and the differences between the means were considered significant when *p* < 0.05. pEC50 values were calculated through non-linear regression (Neubig et al., 2003), and Emax was obtained by averaging the maximum percentages of contraction or relaxation. All results were analyzed with the GraphPad Prism^®^ version 5.01 (GraphPad Software Inc., San Diego CA, United States).

## Results

Effect of supplementation with S. platensis on the contractile response induced by CCh in the presence of functional epithelium.

**FIGURE 1:**
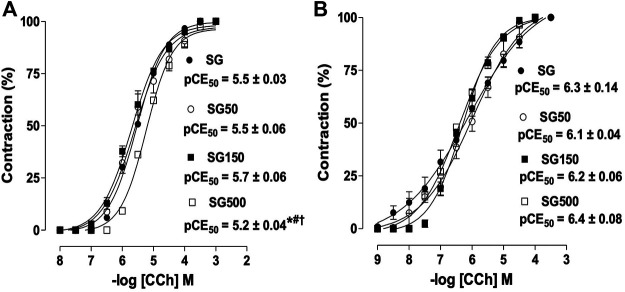
Contractile effect of CCh in SG (●), SG50 (○), SG150 (■) and SG500 groups (.) in rat trachea in the presence **(A)** and absence of epithelium **(B)**. The symbols and vertical bars represent the mean and S.E.M., respectively (n = 5). One-way ANOVA followed by Bonferroni’s post-test. *p < 0.05 (SG *vs.* SG500), ^#^ *p* < 0.05 (SG50 *vs.* SG500) and ^†^ *p* < 0.05 (SG150 *vs.* SG500). CCh = carbachol. Healthy saline group = SG. Healthy group supplemented with *S. platensis* at 50, 150 and 500 mg/kg = SG50, SG150 and SG500, respectively.

Among the healthy groups treated with S. platensis, the 500 mg/kg dose shifted the cumulative concentration-response curve of CCh to the right (pEC50 = 5.2 ± 0.06) compared to SG, SG50, and SG150 (pEC50 = 5.5 ± 0.03, 5.5 ± 0.06 and 5.7 ± 0.06, respectively) in the presence of epithelium (Figure 1A). Contractile response induced by CCh in rat trachea without functional epithelium did not change by the supplementation with *S. platensis* (Figure 1B).

Effect of supplementation with S. platensis on the relaxation induced by aminophylline.

**FIGURE 2:**
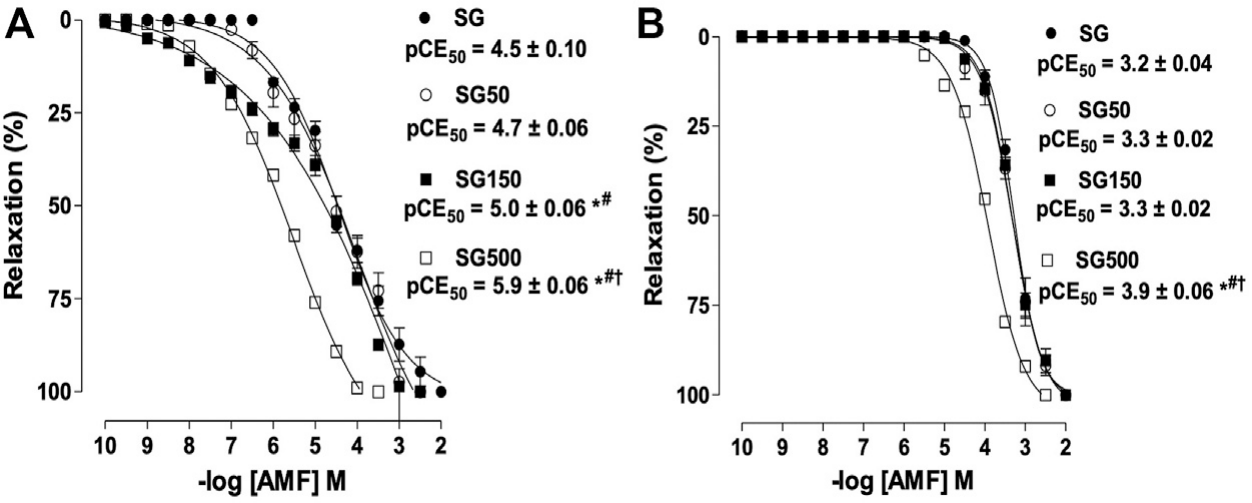
The relaxant effect of AMF on the tonic contractions induced by CCh in SG (●), SG50 (○), SG150 (■) and SG500 groups (.) in rat trachea in the presence **(A)** and absence of epithelium **(B)**. The symbols and vertical bars represent the mean and S.E.M., respectively (n = 5). One-way ANOVA followed by Bonferroni’s post-test. *p < 0.05 (SG *vs.* SG150 and SG500), ^#^ *p* < 0.05 (SG50 *vs.* SG150 and SG500) and ^†^ *p* < 0.01 (SG150 *vs.* SG500). AMF = aminophylline. Healthy saline group = SG. Healthy group supplemented with *S. platensis* at 50, 150 and 500 mg/kg = SG50, SG150 and SG500, respectively.

The relaxing potency of aminophylline increased significantly when the healthy animals received supplementation with *S. platensis* at 150 (pEC50 = 5.0 ± 0.05) and 500 mg/kg (pEC50 = 5.9 ± 0.06) when compared to SG and SG50 (pEC50 = 4.5 ± 0.10 and 4.5 ± 0.02, respectively), but we did not observe changes in Emax (Figura 2A). The relaxing potency of aminophylline in rat trachea without functional epithelium also increased significantly for the group supplemented with S. platensis at 500 mg/kg when compared to healthy animals supplemented with S. platensis at doses of 50 and 150 mg/kg (pEC50 = 4.0 ± 0.04 vs. 3.4 ± 0.04, 3.4 ± 0.02, 3.3 ± 0.02, respectively) (Figure 2B).

Effect of supplementation with S. platensis on the cumulative contractions induced by CCh in the absence and presence of indomethacin.

**FIGURE 3:**
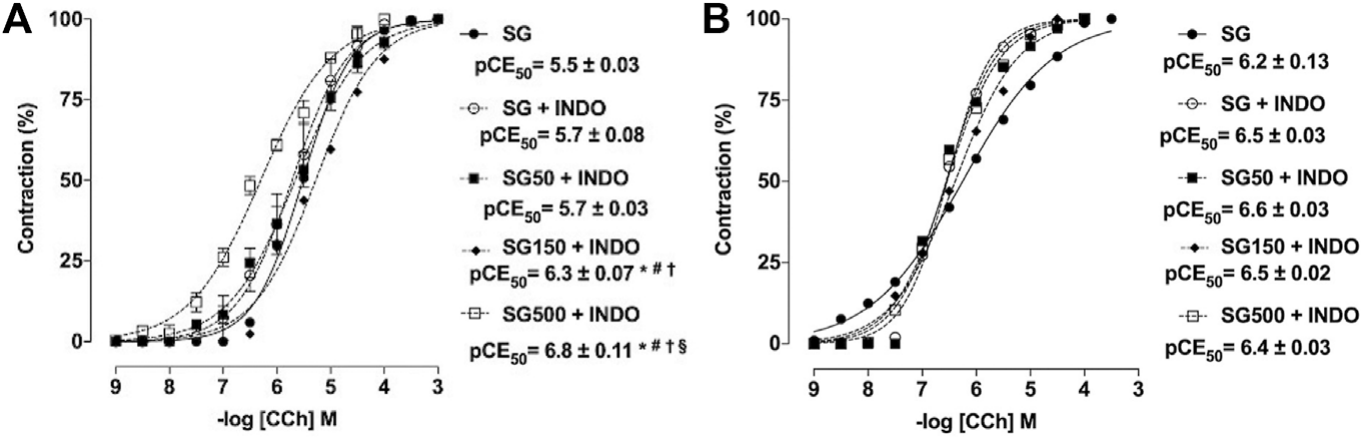
Contractile effect of CCh in the absence SG (●) and presence of indomethacin in SG50 (fx1), SG50 (■) and SG150 (fx2) SG500 groups (fx3) in rat trachea in the presence **(A)** and absence of epithelium **(B)**. The symbols and vertical bars represent the mean and S.E.M., respectively (n = 5). One-way ANOVA followed by Bonferroni’s post-test. *p < 0.05 (SG *vs.* SG150 + INDO and SG500 + INDO), ^#^ *p* < 0.05 (SG + INDO *vs.* SG150 + INDO and SG500 + INDO), ^†^ *p* < 0.05 (SG50 + INDO *vs.* SG150 + INDO and SG500 + INDO) and ^§^ *p* < 0.05 (SG150 + INDO *vs.* SG500 + INDO). INDO = indomethacin. Healthy saline group = SG. Healthy group supplemented with *S. platensis* at 50, 150 and 500 mg/kg = SG50, SG150 and SG500, respectively.

In the presence of indomethacin (INDO), the cumulative concentration-response curve to CCh shifted to the left for the healthy animals supplemented with *S. platensis* at 150 and 500 mg/kg when compared to SG and SG50 in the presence of INDO (pEC50 = 6.3 ± 0.07 and 6.8 ± 0.11 vs. 5.7 ± 0.08 and 5.7 ± 0.08, respectively) (Figure 3A). The contractile response induced by CCh in rat trachea without functional epithelium did not change with supplemented with *S. platensis* in healthy animals in the presence of INDO (Figure 3B).

Effect of supplementation with S. platensis on nitrite production in plasma and trachea rings.

**FIGURE 4:**
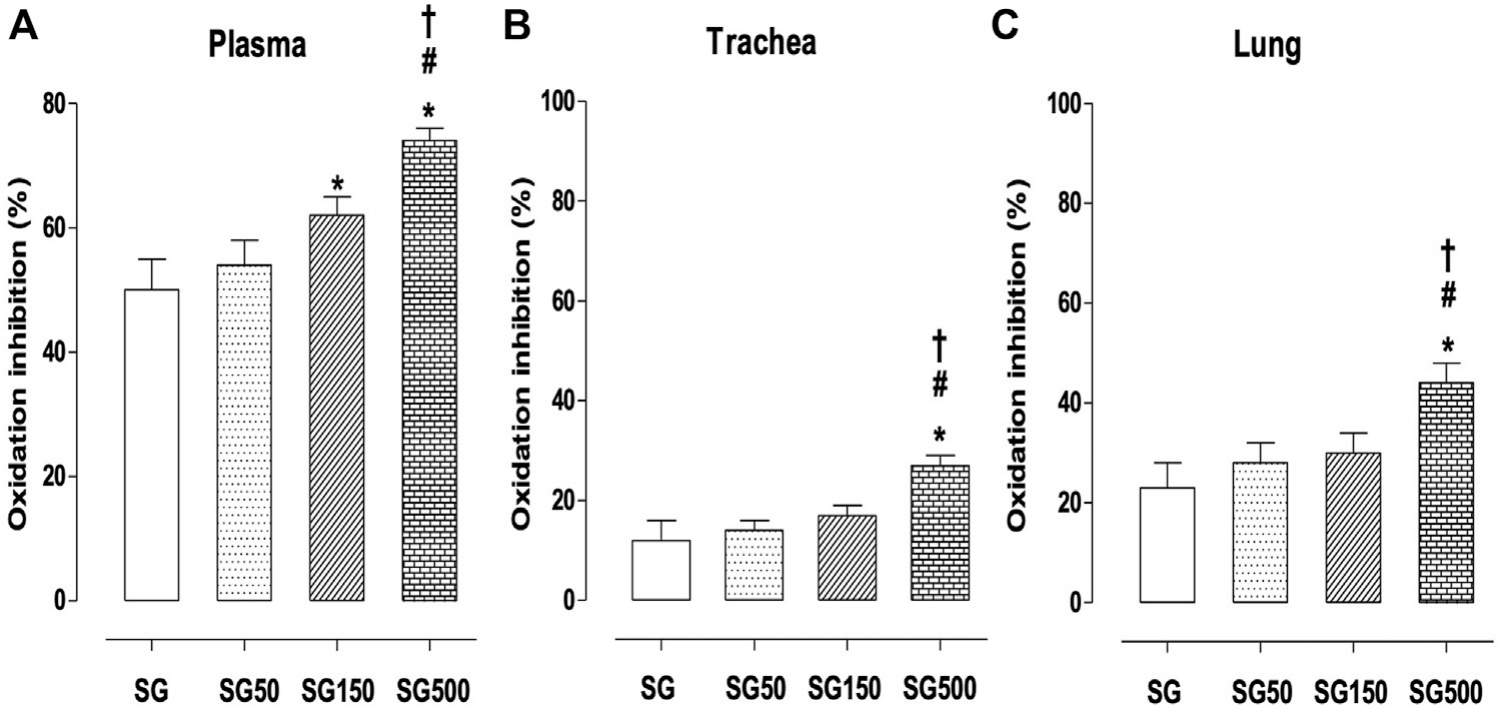
Concentration of nitrite in blood plasma **(A)**, trachea **(B)** and lung **(C)** from SG (fx4), SG50 (fx5), SG150 (fx6), and SG500 groups (fx7). The columns and vertical bars represent the mean and S.D., respectively (n = 10). One-way ANOVA followed by Bonferroni’s post-test. *p < 0.05 (SG *vs*. SG150 and SG500), ^#^ *p* < 0.05 (SG50 *vs.* SG500) and ^†^ *p* < 0.05 (SG150 *vs.* SG500). Healthy saline group = SG. Healthy group supplemented with *S. platensis* at 50, 150 and 500 mg/kg = SG50, SG150, and SG500, respectively.

Nitrite concentration on plasma was higher with supplementation of *S. platensi*s at all doses tested, 50, 150, and 500 mg/kg. Values increased from 54.0 ± 11.0 µM (SG) to 70.0 ± 7.0 (SG150) and 88.0 ± 7.0 µM (SG500) (Figure 4A). Analyzing the production of nitrite in the trachea, when comparing the healthy (34.0 ± 10.0 µM) and supplemented groups, we only observed the increase in nitrite levels in the group with supplementation of *S. platensis* at 500 mg/kg (54.0 ± 8.0 µM), also when compared to SG50 (37.0 ± 10.0 µM) and SG150 (38.0 ± 7.0 µM) (Figure 4B).

Effect of supplementation with S. platensis on MDA production in plasma, trachea rings, and lung.

**FIGURE 5:**
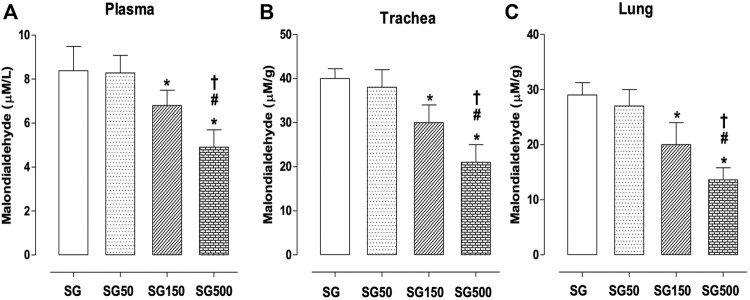
Lipid peroxidation in blood plasma **(A)**, trachea **(B)**, and lung **(C)** from SG (fx4), SG50 (fx5), SG150 (fx6), and SG500 groups (fx7). The columns and vertical bars represent the mean and S.D., respectively (n = 10). One-way ANOVA followed by Bonferroni’s post-test. *p < 0.05 (SG *vs*. SG150 and SG500), ^#^ *p* < 0.05 (SG50 *vs*. SG150 and SG500) and ^†^ *p* < 0.05 (SG150 *vs*. SG500). Healthy saline group = SG. Healthy group supplemented with *S. platensis* at 50, 150, and 500 mg/kg = SG50, SG150, and SG500, respectively.

The concentration of MDA in plasma was lower in healthy animals with supplementation of *S. platensis* at doses of 150 and 500 mg/kg. The values decreased from 8.3 ± 1.1 nmol/L (SG) to 6.8 ± 0.7 (SG150) and 5.0 ± 0.1 nmol/L (SG500) (Figure 5A). Furthermore, we observed a reduction in MDA production in the trachea of rats that received supplementation with *S. platensis* at doses of 150 and 500 mg/kg. Values decreased from 40.0 ± 5.0 μM/g (SG) to 29.0 ± 4.0 (SG150) and 21.0 ± 4.0 μM/g (SG500) (Figure 5B). Similarly, in the lung, we found a decrease in MDA concentration when comparing SG (29.0 ± 5.0 μM/g) to SG150 (20.0 ± 4.0 μM/g) and SG500 (14.0 ± 2.0 μM/g) (Figure 5C).

Effect of supplementation with S. platensis on antioxidant activity in plasma, trachea rings, and lung.

**FIGURE 6:**
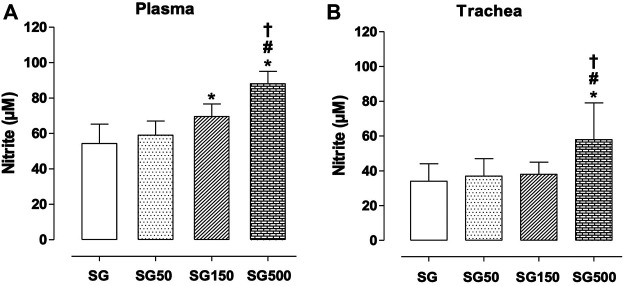
Percentage of oxidation inhibition in blood plasma **(A)**, trachea **(B)** from SG (fx4), SG50 (fx5), SG150 (fx6), and SG500 groups (fx7). The columns and vertical bars represent the mean and S.D., respectively (n = 10). One-way ANOVA followed by Bonferroni’s post-test. *p < 0.05 (SG *vs*. SG150 and SG500), ^#^ *p* < 0.05 (SG50 *vs*. SG500) and ^†^ *p* < 0.05 (SG150 *vs*. SG500). Healthy saline group = SG. Healthy group supplemented with *S. platensis* at 50, 150, and 500 mg/kg = SG50, SG150, and SG500, respectively.

In groups subjected to supplementation with *S. platensis* at doses of 150 and 500 mg/kg, we found a higher percentage of inhibition of plasma oxidation when compared to healthy animals. The values increased from 50.0 ± 5.0% (SG) to 62.0 ± 3.0 (SG150) and 74.0 ± 2.0% (SG500) (Figure 6A). In the trachea, the healthy animals showed an increased percentage of oxidation inhibition with the supplementation of *S. platensis* at 500 mg/kg. The values increased from 8.0 ± 4.0% (SG) to 26.0 ± 2.0% (SG500) (Figure 6B). Similarly, in the lung, we found an increase in oxidation inhibition when comparing SG (23.0 ± 7.0%) to SG150 (30.0 ± 3.0%) and SG500 (44.0 ± 2.0%) (Figure 6C).

## Discussion

In the present study, we tested the influence of different doses of Spirulina platensis on the tracheal reactivity of healthy animals. Our main findings are that the doses of 150 and 500 mg/kg of this cyanobacterium promote increase in tracheal rings relaxation. While the dose of 500 mg/kg promote a decrease in contractile potency and an increase in tracheal rings relaxation. Indicating that in healthy animals higher doses are needed to promote prophylactic effects on physiological responses. In addition, there was a significant contribution of *S. platensis* in the increase in nitrite concentration and the decrease in oxidative stress.

Here, carbachol worked as an inducer of cumulative contraction curves in tracheal rings. The results obtained for the control group demonstrated pEC50 values similar to those verified in previous research by Brito et al. (2015) and Denis et al. (2001). Added to this, Albuquerque et al. (Albuquerque et al., 2016) report that the use of carbachol for contractions induction in tracheal rings was widely verified and accepted to study the reactivity of the trachea as a mimic of cholinergic stimulation.

Albuquerque et al. (2016) also reported that the contraction of trachea smooth muscle has similar mechanisms to those found in the smooth muscle of blood vessels. Thus, the association of agonists with G protein (heterotrimeric complex with α, β, and γ subunits) catalyzes the breakdown of phosphatidylinositol 4,5-bisphosphate resulting in the formation of two intracellular messengers: diacylglycerol and inositol 1,4,5-triphosphate, which promotes an intracellular release of calcium (Juárez-Oropeza et al., 2009). In contrast, the data in the literature have shown that *S. platensis* reduces the contractile potential of aortic rings (Juárez-Oropeza et al., 2009; Albuquerque et al., 2016). Briefly, Mascher et al. (2006) and Juárez-Oropeza et al. (2009) demonstrated that administration of *S. platensis*, in a dose-dependent manner and in obese animals, produced changes in vasomotor reactivity, which led to a decrease in the contractile response to phenylephrine and maximum stress and promoted dose-dependent relaxation in aortic rings. Both authors further suggest that, in addition to *S. platensis* being strongly related to the mechanisms of synthesis and release of nitric oxide by the endothelium in these rings, it also appears to inhibit the process of synthesis/release of a vasodilator dependent on the cyclooxygenase pathway.

From the research done by Mascher et al. (2006) and Juárez-Oropeza et al. (2009) it is noteworthy that the data result from investigations on aortic rings, while we are the pioneers in demonstrating that the tracheal rings of animals submitted to supplementation with *S. platensis* also present lower contractile potency and greater relaxation potential. Thus, the data presented in this investigation corroborate with those previously found but on different physiological systems.

In allergic diseases of the respiratory tract, it is well established in the literature that asthma and allergic rhinitis share the same physiological substrate: the trachea. Therefore, contractions in the smooth muscle of this region trigger different responses according to the disease, for the first one is bronchial hyperreactivity, while the second is upper airway obstruction (Fahy, 2015; Zhong et al., 2018). In this study, we verified the effectiveness of non-drug therapies, such as *Urtica dioica*, *Sambucus nigra*, and *Spirulina platensis*, in the prevention or treatment of contractile reactivity of the trachea (Ibrahim et al., 2013).

The literature shows that the contractile reactivity of the trachea in respiratory allergic diseases is accompanied by an increase in inflammatory markers such as interleukin-4 and -5 and tumor necrosis factor-α (TNF-α) (Joskova et al., 2013; Franova et al., 2016). However, until the present study, it was still not possible to identify whether *S. platensis* would also influence markers of vascular relaxation and oxidative stress such as nitric oxide and malondialdehyde, respectively, on tracheal rings in healthy animals. We based this hypothesis on the fact that C-phycocyanins, active ingredient of S. platensis, and natural dyes used in the pharmaceutical or nutritional industry (Abalde et al., 1998) selectively inhibit the activity of cyclooxygenase-2, the enzyme responsible for the production of prostaglandins, in addition to their chemical structure possessing antioxidant property functioning as a scavenger of free radicals (Reddy et al., 2000). It was also observed that the high dose of S. platensis actually increased nitrite levels, and that despite it being a precursor to nitrosative stress, concomitantly for the same supplementation doses, we also identified a high rate of inhibition of oxidation in the trachea, ratifying the antioxidant power of Spirulina platensis. We, in this paper, demonstrated that S. platensis positively modulates the production of nitrite, which is the end product of nitric oxide metabolism, increases inhibition of oxidation, and reduces lipid peroxidation in tracheal rings.

The results of the present study confirm the beneficial effect of the frequent use of Spirulina platensis in the prevention of diseases of the respiratory system. As previously demonstrated, S. platensis has been used in humans’ diets for many years as a food or supplement in the form of powders, beverages, or capsules (Appel et al., 2018); it received worldwide repercussion when the National Aeronautics and Space Administration (NASA) included it as part of the astronaut’s diet both because of its nutritional composition, rich in proteins and minerals, and its anti-inflammatory and antioxidant effects (Karkos et al., 2011; Finamore et al., 2017). Therefore, considering the current scenario with a high incidence of diseases that can affect the respiratory system, having access to a food of ancient use with nutraceutical properties among different ethnic groups is extremely necessary. Despite these findings, it is important to emphasize that our research is a preclinical study. Making further investigation with Spirulina platensis necessary in models that analyze lung function, as well as in standardized models of asthma, is needed to confirm the benefit of algal supplementation on airway reactivity.

## Data Availability

The raw data supporting the conclusions of this article will be made available by the authors, without undue reservation.
